# Evolution of public cooperation in a monitored society with implicated punishment and within-group enforcement

**DOI:** 10.1038/srep17050

**Published:** 2015-11-24

**Authors:** Xiaojie Chen, Tatsuya Sasaki, Matjaž Perc

**Affiliations:** 1 School of Mathematical Sciences, University of Electronic Science and Technology of China, Chengdu 611731, China; 2 Faculty of Mathematics, University of Vienna, 1090 Vienna, Austria; 3 Faculty of Natural Sciences and Mathematics, University of Maribor, SI-2000 Maribor, Slovenia; 4 Department of Physics, Faculty of Sciences, King Abdulaziz University, Jeddah, Saudi Arabia; 5 Center for Applied Mathematics and Theoretical Physics, University of Maribor, SI-2000 Maribor, Slovenia

## Abstract

Monitoring with implicated punishment is common in human societies to avert freeriding on common goods. But is it effective in promoting public cooperation? We show that the introduction of monitoring and implicated punishment is indeed effective, as it transforms the public goods game to a coordination game, thus rendering cooperation viable in infinite and finite well-mixed populations. We also show that the addition of within-group enforcement further promotes the evolution of public cooperation. However, although the group size in this context has nonlinear effects on collective action, an intermediate group size is least conductive to cooperative behaviour. This contradicts recent field observations, where an intermediate group size was declared optimal with the conjecture that group-size effects and within-group enforcement are responsible. Our theoretical research thus clarifies key aspects of monitoring with implicated punishment in human societies, and additionally, it reveals fundamental group-size effects that facilitate prosocial collective action.

Public cooperation is imperative for the sustainable management of common resources in human societies^1^,^2^,^3^. However, human cooperation is threatened by temptations that are rooted in selfish but lucrative short-term benefits on offer when defecting or free-riding on the efforts of others^4^. Like rewarding^5^,^6^,^7^,^8^,^9^, punishment is often employed for maintaining sufficiently high levels of public cooperation^10^,^11^,^12^,^13^,^14^,^15^,^16^,^17^,^18^,^19^. In addition to individual efforts aimed at punishing free-riders^20^,^21^,^22^,^23^,^24^,^25^, our societies are home to a plethora of sanctioning institutions^26^,^27^. In particular, during the last decade peer and pool punishment have been studied theoretically and experimentally as possible means to stabilize cooperation^20^,^21^,^26^,^28^,^29^,^30^,^31^,^32^,^33^,^34^,^35^,^36^.

Although ample research efforts have already been invested to inform on the subtleties of positive and negative reciprocity and their role in promoting public cooperation^11^, few studies have thus far considered implicated punishment despite it being and integral cog in various sanctioning systems in human societies. In general, the implementation of implicated punishment means that once a wrongdoer is caught, all the group members are punished, no matter whether the group members are cooperators or defectors. Such punishment schemes are particularly common for monitoring^37^ the management of common resources on large scales. For example, in Nature Reserve of China, an administrative bureau is responsible for monitoring all illegal activities. When the bureau staff members detect an illegal activity in the monitored parcel, all households within the group will suffer the same fine^14^. While the system may work in practice, in theory it is still unclear how fines affect cooperators that are adversely affected, and how the overall dynamics plays out in favor of prosocial behaviour.

In addition to the well-known and important adverse effects that emerge if cooperators are sanctioned^38^,^39^, some individuals in the group may act emotionally and exploit options related to within-group enforcement^32^,^40^,^41^,^42^, for example resorting to probabilistic peer punishment^43^. It is thus also of interest to consider whether the addition of probabilistic within-group enforcement can further enhance the evolution of cooperation in the presence of monitoring and implicated punishment. In fact, a recent study based on field observations found that an intermediate group size is optimal for public cooperation when both implicated punishment and within-group enforcement are present^14^. However, there is no theoretical research available that would support the conjecture that group-size effects and within-group enforcement are responsible for the success of implicated punishment.

In this paper, we therefore consider a public goods game with implicated punishment and within-group enforcement in infinite and finite well-mixed populations. Our goal is to develop a thorough theoretical understanding behind the success of implicated punishment, and the role within-group enforcement and group size play in either supporting or impairing the evolution of public cooperation. As we will show, implicated punishment transforms the public goods game into a coordination game, and within-group enforcement further promotes the emergence of prosocial collective action. Contrary to field observations^14^, however, theory fails to predict an optimal intermediate group size for the evolution of cooperation. Instead, we find that an intermediate group size is actually not beneficial for the successful evolution of cooperation. Our research thus clarifies key aspects of monitoring with implicated punishment in human societies, and it also reveals fundamental group-size effects that may promote a public agenda.

## Results

We consider a well-mixed population, in which individuals engage in a public goods game^44^, where each individual is able to cooperate or to defect, respectively. In each group, *N* players are chosen randomly to form a group for playing the game. Cooperators contribute the cost *c*, while defectors contribute nothing. The sum of all contributions in the group is multiplied by the enhancement factor *r* > 1, and then split evenly among all group members. After choosing the strategy, the group’s behaviours will be monitored with a probability *p* (0 < *p* < 1). If it is detected that there is at least one defector in the group, then the implicated punishment mechanism will work, and accordingly each individual will incur a fine *d* (*d* > 0). Otherwise, there is no monitoring, and there is no fine on any individual. But once the implicated punishment is implemented in the group, it may trigger the within-group enforcement. Accordingly, each cooperator (if present) will use the peer punishment on defectors with a probability *q* (0 < *q* < 1), and is designated as a punisher. Peer punishers impose a fine *β* on each defector at a cost *α* (0 < *α* < *β*).

Below, we study how the introduction of implicated punishment and within-group enforcement influences the evolutionary dynamics of cooperation both in infinite and finite well-mixed population, in particular the effects of group size in the model, by theoretical and numerical analysis. We emphasize that the social dilemma only exists when *r* < *N* in the public goods game^44^,^45^, so in this study the interval of *r* values is constrained as 1 < *r* < *N*.

**Figure 1:**
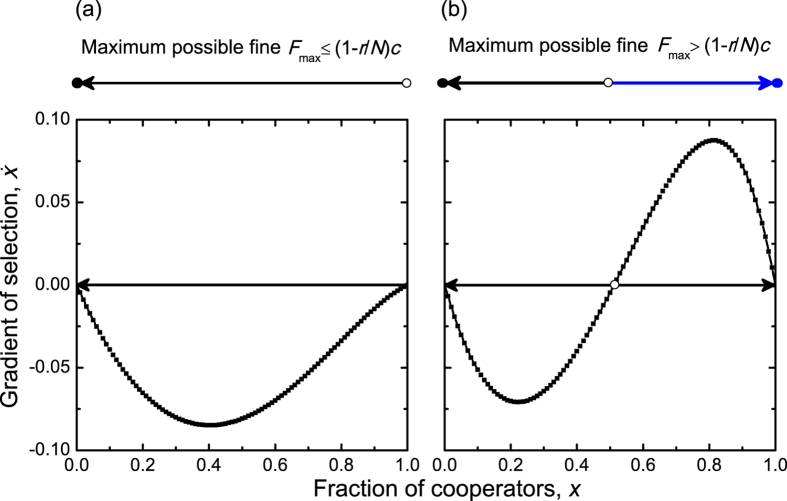
The gradient of selection in dependence on the fraction of cooperators in infinite populations. Stable equilibria are depicted with solid circles, while unstable equilibria are depicted with open circles. Arrows indicate the expected direction of evolution. Cooperation is favored over defection when the arrow points to the right. When the maximal possible average fine for a defector *F*_*max*_ = *dp* + *pq*(*N* − 1)*β* ≤ (1 − *r*/*N*)*c*, the public good dilemma still exists with full defection as the only stable equilibrium (**a**). Otherwise, the public good game is transformed into a coordination game with full cooperation and full defection as the two stable equilibria (**b**). Parameter values are: *N* = 5, *r* = 3, *c* = 1, *d* = 1.0, *p* = 0.1, *α* = 0.3, *β* = 1.0, and *q* = 0.5 in (**a**); *N* = 5, *r* = 3, *c* = 1, *d* = 1.0, *p* = 0.5, *α* = 0.3, *β* = 1.0, and *q* = 0.5 in (**b**).

We first present the gradient of selection 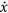 given by the replicator equation (Methods for infinite populations) for studying the evolution of cooperative behaviour in infinite populations, as illustrated in Fig. 1. Here, *x* is the fraction of all the cooperators in the infinite population. We show that there exist two typical behaviours for the gradient of selection varying with the fraction of cooperators, as presented in Fig. 1(a,b) respectively. We define *F*_*max*_ as the maximal fine upon a defector who receives from the two punishment regimes, *dp* + *pq*(*N* − 1)*β*. We accordingly prove that if *F*_*max*_ ≤ (1 − *r*/*N*)*c* (Methods for infinite populations), the gradient of selection is always negative (Fig. 1(a)). Cooperators thus die out regardless of the initial conditions. While if *F*_*max*_ > (1 − *r*/*N*)*c*, a new unstable equilibrium emerges in the *x* ∈ (0, 1) interval, which divides the system into two basins of attraction (Fig. 1(b)). Depending on the initial conditions, thus the system will evolve either towards full defection or towards full cooperation. Both *x* = 0 and *x* = 1 are stable steady states, indicating that the public goods game is transformed into a coordination game.

**Figure 2:**
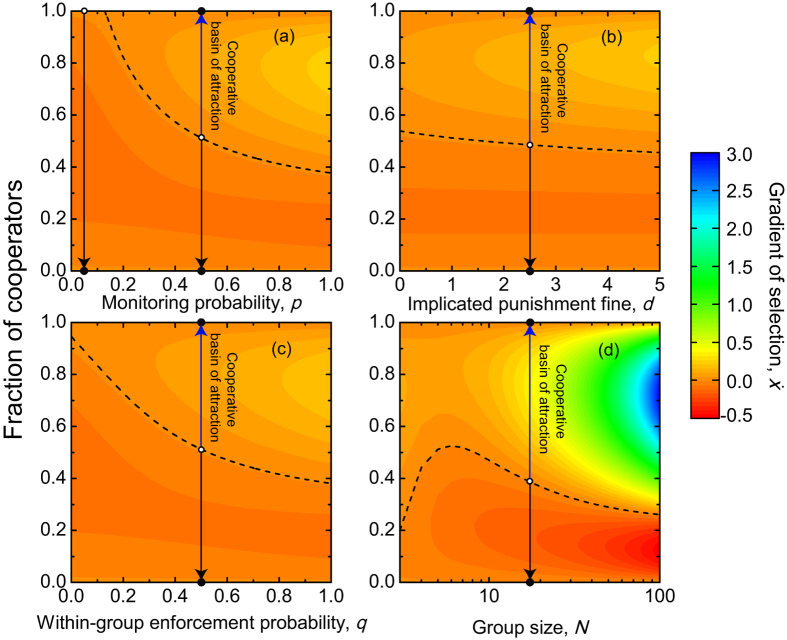
The stationary fraction of cooperators and the gradient of selection in infinite populations. The unstable internal equilibrium (if present) dividing the system into two basins of attraction is indicated by dash lines and also by open circles, and the cooperative basin of attraction is indicated by blue arrows. The stable boundary equilibrium is indicated by solid circles, while the unstable boundary equilibrium is indicated by open circles. The gradient of selection in the areas above the dash line is positive, while the gradient of selection in the areas below the dash line is negative. And the magnitude of the gradient of selection is shown using the red-green-blue scale indicated, and blue areas indicate parameter combination for which the fraction of cooperators increases faster. In (**a**–**c**), the unstable internal equilibrium decreases with increasing the monitoring probability *p*, the implicated punishment fine *d*, and the within-group enforcement probability *q*, respectively. While in (**d**) the unstable internal equilibrium first increases, then decreases with increasing the group size *N*. In other words, increasing *p*, *d*, or *q* enlarges the basin of attraction of the *x* = 1 stable state, thus favoring the evolution of cooperation. Importantly, a small group size or a large group size can lead to a larger basin of attraction of the *x* = 1 stable state than an intermediate group size does. In addition, in the areas above the dash lines, the rate of increase of the fraction of cooperators depends on the parameter values. Parameter values are: *N* = 5, *r* = 3, *c* = 1, *d* = 1.0, *α* = 0.3, *β* = 1.0, and *q* = 0.5 in (**a**); *N* = 5, *r* = 3, *c* = 1, *p* = 0.5, *α* = 0.3, *β* = 1.0, and *q* = 0.5 in (**b**); *N* = 5, *r* = 3, *c* = 1, *p* = 0.5, *d* = 1.0, *α* = 0.3, and *β* = 1.0 in (**c**); *r* = 3, *c* = 1, *p* = 0.5, *d* = 1.0, *α* = 0.3, *β* = 1.0, and *q* = 0.5 in (**d**).

Furthermore, we investigate how the parameters influence the stationary fraction of cooperators in the infinite population, as shown in Fig. 2. We find that when the monitoring probability *p* is zero or small, there is always no interior equilibrium, regardless of the values of other parameters in Fig. 2(a). When *p* increases to *c*(1 − *r*/*N*)/[*d* + *q*(*N* − 1)*β*] (Methods for infinite populations), an interior equilibrium which is unstable enters the state space at the point *x* = 1. With further increasing *p*, the interior equilibrium decreases. In other words, increasing the monitoring probability enlarges the basin of attraction of the *x* = 1 steady state. We now consider the effects of implicated fine *d*. When *d* = 0, if *pq*(*N* − 1)*β* > *c*(1 − *r*/*N*), then there is an interior equilibrium (Methods for infinite populations). Otherwise, no interior equilibrium can emerge. If the interior equilibrium is present, it decreases with increasing *d* (Fig. 2(b)), which means that increasing the implicated fine *d* also enlarges the basin of attraction of the *x* = 1 steady state. It is necessary to point out that compared to the increase of *p*, the increase of *d* makes the value of the interior equilibrium decrease much slowly. This means that the chance of monitoring can result in more positive effects on the evolution of cooperation than the punishment fine does, when the probabilistic implicated punishment is considered. In addition, when the probability for within-group enforcement *q* is zero, the interior equilibrium presents if *dp* > *c*(1 − *r*/*N*) (Methods for infinite populations). Then it decreases with increasing *q*, accordingly the basin of attraction of the *x* = 1 steady state is enlarged (Fig. 2(c)). Finally, we investigate the effects of group size *N*. Interestingly, we find that if the interior equilibrium is present, it first increases, reaches a maximum, but then decreases with increasing the group size (Fig. 2(d)). This means that the basin of attraction of the *x* = 1 steady state is smallest at an intermediate group size. We also find that the interior equilibrium could be absent for small group size, depending on the values of other parameters. But it will exhibit then when the group size increases to a certain value (Supplementary Fig. S1). Subsequently, the interior equilibrium decreases with further increasing the group size, which indicates that the larger the group size, the greater the basin of attraction of *x* = 1. This finding is in agreement with previous experimental results in^46^. Furthermore, we emphasize that no matter how large the values of *p*, *d*, *q*, and *N* are, the boundary equilibrium *x* = 0 is always stable, which means that the outcome that *x* = 1 is the only stable state cannot happen in our model (Methods for infinite populations).

It is worth pointing out that in line with Ref. 14, group size is found to be able to produce nonlinear effects on collective action in our study. But being contrary to the field observation, we find that an intermediate group size cannot lead to the most favorable outcome for public cooperation. Instead, it could lead to the smallest basin of attraction of the full cooperation state, which indicates that an intermediate group size is not beneficial to the evolution of cooperation when the implicated punishment and within-group enforcement are incorporated.

In addition, we show the gradient of selection 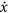 in Fig. 2, and indicate that its value in the areas above the dash line is positive. If the gradient of selection is positive, the fraction of cooperators will increase. We see that with increasing the monitoring probability *p*, the implicated punishment fine *d*, or the within-group enforcement probability *q*, the gradient of selection increases in the ares where 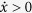. However, in that area the gradient of selection first decreases, reaches a minimum, but then increases with increasing the group size. For a fixed value of *p*, *d*, *q*, or *N*, the gradient of selection can always reach the maximal values at an intermediate fraction of cooperators, which is smaller than *x* = 1.

**Figure 3:**
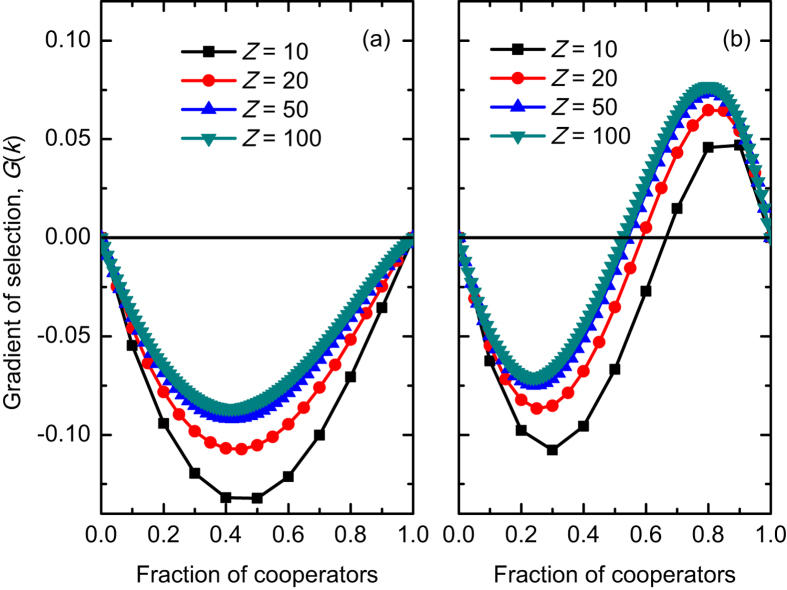
The gradient of selection in dependence on the fraction of cooperators in finite populations for different population size. The gradient of selection exhibits two qualitatively identical behaviours as reported in Fig. 1. Parameter values are: *N* = 5, *r* = 3, *c* = 1, *p* = 0.1, *d* = 1.0, *α* = 0.3, *β* = 1.0, and *q* = 0.5 in (**a**); *N* = 5, *r* = 3, *c* = 1, *p* = 0.5, *d* = 1.0, *α* = 0.3, *β* = 1.0, and *q* = 0.5 in (**b**).

Corresponding to the right-hand side of the replicator equation, we use the gradient of selection *G*(*k*)^47^,^48^ (Methods for finite populations), to describe the behavioural dynamics in finite populations. Figure 3 shows two typical behaviours of *G*(*k*) as a function of the fraction of cooperators *k*/*Z* for different sizes *Z* of finite populations. We find that the two typical behaviours found in infinite populations are also valid in finite populations, for any parameter combinations. One behaviour depicts that *G*(*k*) < 0 for any *k*, which shows that cooperators are always disadvantageous. The other depicts that *G*(*k*) has a unique internal root k^*^, above which *G*(*k*) > 0. This means that cooperators become advantageous when *k* is larger than k^*^. In addition, with increasing the population size, the gradient of selection increases. This results in that the position of the interior root moves from right to left by increasing the population size. Thus, the range of *k*/*Z* in which cooperators are advantageous is greatly increased for large populations.

**Figure 4:**
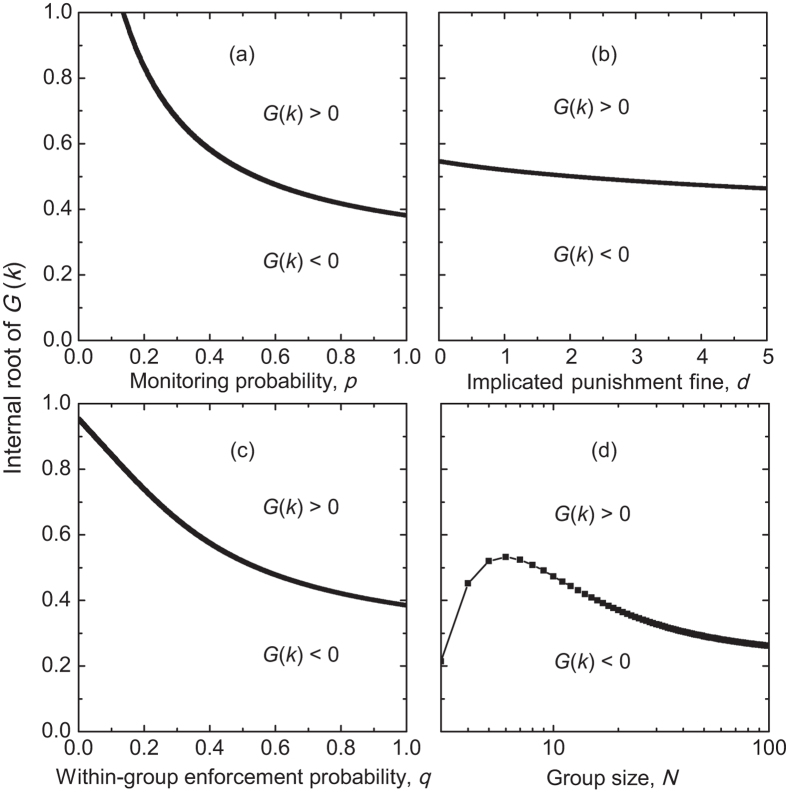
The internal roots of the gradient of selection *G*(*k*) in finite populations. The roots are normalized by the population size *Z*. In (**a**–**c**), the values of roots decrease with increasing the monitoring probability *p*, the fine of implicated punishment *d*, and the probability of within-group enforcement *q*, respectively. While in (**d**), the values first increase, and then decrease with increasing the group size *N*. Parameter values are: *Z* = 200, *r* = 3, *c* = 1, *N* = 5, *d* = 1.0, *α* = 0.3, *β* = 1.0, and *q* = 0.5 in (**a**); *Z* = 200, *r* = 3, *c* = 1, *N* = 5, *p* = 0.5, *α* = 0.3, *β* = 1.0, and *q* = 0.5 in (**b**); *Z* = 200, *r* = 3, *c* = 1, *N* = 5, *p* = 0.5, *α* = 0.3, *β* = 1.0, and *d* = 1.0 in (**c**); *Z* = 200, *r* = 3, *c* = 1, *p* = 0.5, *d* = 1.0, *α* = 0.3, *β* = 1.0, and *q* = 0.5 in (**d**).

In what follows, we show how the interior root of *G*(*k*) varies with the parameters which have been referred to infinite populations (Fig. 4). We find that when the root exists, its value monotonically decreases with increasing the monitoring probability *p*, the implicated sanction fine *d*, or the within-group enforcement probability *q* (Supplementary Fig. S2(a–c)). This means that the range of *k*/*Z* for which cooperation is advantageous increases when any one of these three parameters (*p*, *d*, and *q*) increases. It is worth pointing out that the value of the interior root decreases much slowly as the implicated punishment fine *d* increases, and this phenomenon is also found in infinite populations indicating that the punishment fine can only provide limited positive effects on cooperation. While with increasing the group size, the root’s value first increases, reaches a maximum, but then decreases again (Fig. 4(d)). This means that the range of *k*/*Z* for which cooperation is advantageous reaches the minimal value at an intermediate group size. However, the root may be present only when the group size exceeds a certain value for other parameter values (Supplementary Fig. S2(d)). With further increasing the group size, the root monotonically decreases. Accordingly, the obtained results in finite population confirm that an intermediate group size is not optimal for the evolution of cooperation, but it is certainly not detrimental for cooperation. This in turn indicates that the combined effects of free-riding and within-group enforcement do not lead to an optimal intermediate group size, contrary to the conjecture in Ref. 14. In addition, we emphasize that the root’s value recovers to that in Fig. 2 when *Z* → +∞, and the dependence of the root’s value on these parameters (*p*, *d*, *q*, and *N*) is very similar to those in infinite well-mixed populations.

**Figure 5:**
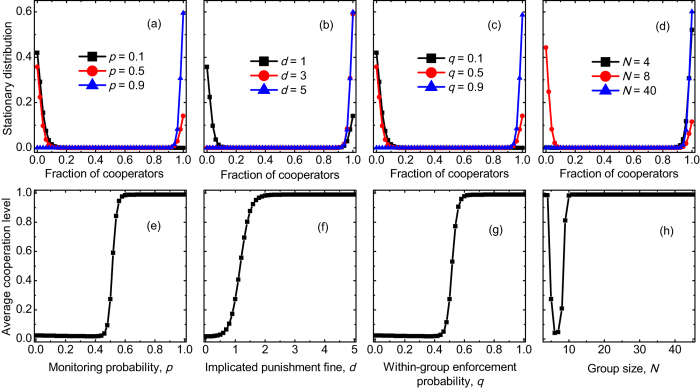
The stationary distribution and the average cooperation level. Top row depicts the stationary distribution in finite populations in the presence of mutation *u* = 0.01. In (**a**–**c**), the population spends more time in states where cooperators prevail for a larger monitoring probability *p*, a larger fine of implicated punishment *d*, or a larger probability of within-group enforcement *q*. While in (**d**) the population spends more time in states where cooperators thrive for either a small group size or a large group size, and spends less time in states where cooperators decline for an intermediate group size. Bottom row depicts the average value of cooperation level in the presence of mutation *u* = 0.01. In (**e**–**g**), the average cooperation level increases with increasing the monitoring probability *p*, the fine of implicated punishment *d*, or the probability of within-group enforcement *q*. While in (**h**), the average cooperation level reaches a high value at either a small group size or a large group size, and reaches a minimum at an intermediate group size. Parameter values are: *Z* = 50, *r* = 3, *c* = 1, *N* = 5, *d* = 1.0, *α* = 0.3, *β* = 1.0, and *q* = 0.5 in (**a, e**); *Z* = 50, *r* = 3, *c* = 1, *N* = 5, *p* = 0.5, *α* = 0.3, *β* = 1.0, and *q* = 0.5 in (**b, f**); *Z* = 50, *r* = 3, *c* = 1, *N* = 5, *p* = 0.5, *α* = 0.3, *β* = 1.0, and *d* = 1.0 in (**c, g**); *Z* = 50, *r* = 3, *c* = 1, *p* = 0.5, *d* = 1.0, *α* = 0.3, *β* = 1.0, and *q* = 0.5 in (**d, h**).

Another key quantity for describing the evolutionary dynamics in finite well-mixed populations is the stationary distribution in the presence of mutations (Methods for finite populations)^49^,^50^. In the top row of Fig. 5, we show how the stationary distribution changes with the four parameters (*p*, *d*, *q*, and *N*), respectively. It is worth pointing out that the stationary distribution characterizes the pervasiveness in time of a given configuration of the population. We find that with increasing the monitoring probability *p*, the implicated sanction fine *d*, or the within-group enforcement probability *q*, the time that the system spends in the full cooperation state increases. With the large values of these parameters, the system spends most of the time in the full cooperation state, leading to maxima of the stationary distribution at *k* = *Z*. But the time that the system spends in the full cooperation state does not monotonically increase with increasing the group size. Instead, with an intermediate group size, the system spends most of the time in the full defection state, leading to maxima of the stationary distribution at *k* = 0. While either a small group size or a large group size leads to that the system spends most of the time in the full cooperation state.

In the bottom row of Fig. 5, we further show how the average value of cooperation level varies with the four parameters (*p*, *d*, *q*, and *N*), respectively. We find that the average cooperation level monotonically increases with increasing the monitoring probability *p*, the implicated sanction fine *d*, or the within-group enforcement probability *q*. But we observe that with increasing the group size, it first decreases, reach a minimum, then increases again. This means that an intermediate group size is not beneficial to the evolution of cooperation. Altogether, Fig. 5 confirms that cooperation is promoted either at a small group size or a large group size, rather than an intermediate group size.

In the Supplementary Information, we also investigate our model in finite populations with large peer punishment cost *α* (Supplementary Fig. S3), and explore the effects of the selection intensity (Supplementary Fig. S4) and the mutation rates (Supplementary Fig. S5) on the stationary distribution of cooperation and the average cooperation level. We find that our results regarding the effects of the monitoring probability, the implicated punishment fine, the within-group enforcement probability, and the group size are not changed when the above variations are considered. In addition, we consider a discounting factor for the implicated punishment fine on cooperators (Supplementary Fig. S6). We find that the introduction of the discounting factor does not change the genetic outcome about the internal root of the gradient of selection in infinite and finite populations, but can decrease the value of the internal root, thus increasing the advantage of cooperators.

## Discussion

Human cooperation is unique, and it is one of the key pillars of our evolutionary success. The origins of our remarkable other-regarding abilities are likely rooted in the mitigation of between-group conflicts, and in the necessity for alloparental care during the advent of the genus *Homo*. In the absence of such pressing challenges, however, human societies rely on rewarding and policing to maintain public cooperation^1^. Monitoring with implicated punishment is a special form of policing, and this form of monitoring and punishment is particularly common. In this paper, based on an evolutionary game theoretical model we have studied the monitoring with implicated punishment and within-group enforcement in infinite and finite well-mixed populations.

As we have emphasized above, our model setup is well aligned with reality in that implicated punishment and within-group enforcement are common in human societies, and it is indeed relatively straightforward to come up with examples where our model could apply. A good example is the large-scale management of common resources in general. The key assumption of implicated punishment is that once a defector within a group is detected, subsequently all members of that group, regardless of their strategies, are fined. Evidently, it is thus likely that cooperators will be punished too. As a countermeasure, we have also considered within-group enforcement through peer punishment. We have shown that the implicated punishment alone transforms the public goods game into a coordination game. Accordingly, cooperation becomes viable, albeit depending somewhat on initial conditions. Adding within-group enforcement to the setup, we have shown that this further relaxes the necessary conditions for coordinated action to emerge, and thus for public cooperation to thrive. Moreover, we have confirmed that cooperation can be enhanced both in infinite and finite well-mixed populations, thus establishing for the first time mechanisms that underlie the success of implicated punishment. Our results also indicate that in the probabilistic implicated punishment the fine has an effect earlier than the monitoring probability for the evolution of cooperation, but before any monitoring benefits materialize a sufficient non-zero punishment fine is required. We hope that this indication about the effects of the punishment fine and the monitoring probability could be helpful for the policy recommendations in the management of common resources.

Since the group size has been identified as a crucial factor affecting collective action^51^,^52^,^53^,^54^,^55^, we have also considered this aspect of the studied evolutionary game in detail. In the typical public goods game, the negative effect of free-riding on cooperation are enhanced by increasing the group size. But when punishment is introduced into the game, it has a positive effect on cooperation especially for large group size^53^. The coexistence of these two opposing factors determines the net effect of the group size, and ultimately the combination of free-riding and punishment leads to the group size having nonlinear effects on collective behaviour. This is in fact predicted quantitatively by our theoretical analysis, and is in agreement with a recent field investigation involving free-riding and within-group enforcement^14^. However, the difference is that our theoretical results show that an intermediate group size is not best for cooperative behaviour, while the field data show the opposite. Importantly, while the conclusions of the field investigation rely solely on the effects of free-riding and within-group enforcement, and also because the range of the available group sizes in the field data was small^14^, the two opposing factors predicted by our theoretical analysis could not have been taken into account. Our study thus provides further key insights on the intricate interplay between the group size, within-group enforcement, and implicated punishment. We hope that our in part counterintuitive results will inspire further theoretical and empirical research devoted to the mechanisms that are essential for prosocial collective behaviour.

## Methods

### Evolutionary dynamics in infinite well-mixed populations

For studying the evolutionary dynamics in infinite well-mixed populations, we use the replicator equation^56^. To begin, we assume a large population, a fraction *x* of which is composed of cooperators, the remaining fraction (1 − *x*) being defectors. Accordingly, the replicator equation is


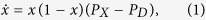


where *P*_*X*_ = *qP*_*P*_ + (1 − *q*)*P*_*C*_ is the average payoff of all the cooperators, while *P*_*P*_, *P*_*C*_, and *P*_*D*_ are the average payoffs of punishing cooperators, second-order free riders (cooperators who do not punish), and defectors, respectively. And the average payoffs *P*_*C*_, *P*_*P*_, and *P*_*D*_ are respectively


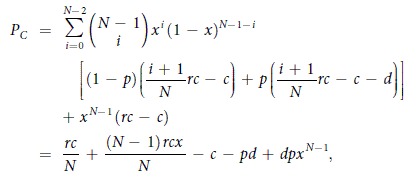



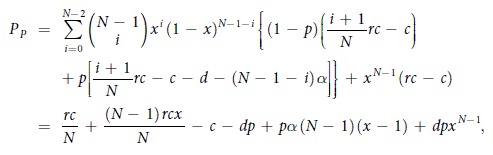


and


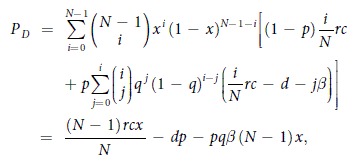


where *i* denotes the number of all the cooperators among *N* − 1 co-players in a group, and *j* (*j* ≤ *i*) denotes the number of punishing cooperators among *i* cooperators.

With these definitions, the replicator equation has two boundary equilibria, namely *x* = 0 and *x* = 1. Interior equilibria, on the other hand, can be determined by the roots of the function *g*(*x*) = *P*_*X*_ − *P*_*D*_, thus obtaining


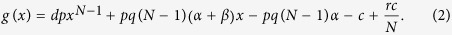


It follows that 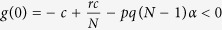 when *r* < *N*. On the other hand, the function *g*(*x*) is strictly increasing since *g*′(*x*) = *dp*(*N* − 1)*x*^*N*−2^ + *pq*(*N* − 1)(*α* + *β*) > 0 for *x* ∈ (0, 1). Accordingly, the interior equilibrium is determined by 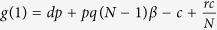, from which we have the following two conclusions:

1. When 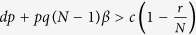, the replicator equation has only one interior equilibrium x^*^ ∈ (0, 1), but it is unstable since *g*′(x^*^) > 0. The two boundary equilibria *x* = 0 and *x* = 1 are both stable.
2. When 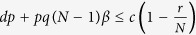, the replicator equation has no interior equilibria in (0, 1). Only *x* = 0 is a stable equilibrium, while *x* = 1 is an unstable equilibrium.

### Evolutionary dynamics in finite well-mixed populations

For studying the evolutionary dynamics in finite well-mixed populations, we consider a population of finite size *Z*. Here, the average payoffs of second-order free-riders, punishing cooperators, and defectors in the population with *k* cooperators are respectively given by


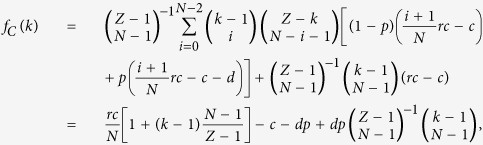



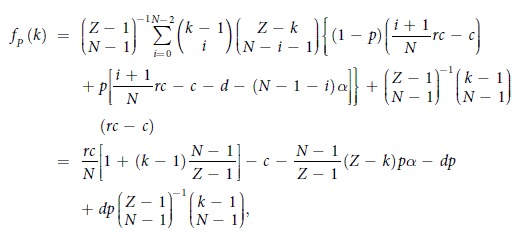


and


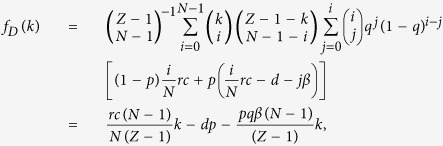


where we impose that the binomial coefficients satisfy 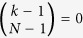 if *k* < *N*.

Consequently, the average payoff of all the cooperators is


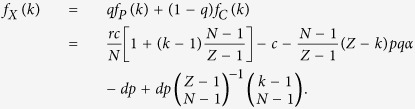


Next, we adopt the pair-wise comparison rule to study the evolutionary dynamics, based on which we assume that player *y* adopts the strategy of player *z* with a probability given by the Fermi function


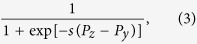


where *s* is the intensity of selection that determines the level of uncertainty in the strategy adoption process^45^,^57^. Without loosing generality, we use *s* = 2.0 in Fig. 5, Supplementary Fig. S3, and Supplementary Fig. S5.

With these definitions, the probability that the number of cooperators in the population increases or decreases by one is


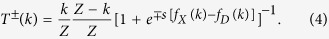


In finite populations, the gradient of selection for arbitrary *s* is thus given by


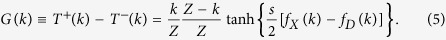


We further introduce the mutation-selection process into the update rule by assuming that mutations occur between cooperators and defectors with probability *μ* in each update step^49^,^58^, and compute the stationary distribution as a key quantity that determines the evolutionary dynamics in finite well-mixed populations. We note that, in the presence of mutations, the population will never fixate in any of the two possible absorbing states. Thus, the transition matrix of the complete Markov chain is


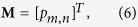


where *p*_*m,n*_ = 0 if |*m* − *n*| > 1, *p*_*m,m*+1_ = (1 − *μ*)*T*^+^(*m*) + *μ*(*Z* − *m*)/*Z*, *p*_*m,m*−1_ = (1 − *μ*)*T*^−^(*m*) + *μm*/*Z*, and *p*_*m,m*_ = 1 − *p*_*m,m*+1_ − *p*_*m,m*−1_ otherwise. Accordingly, the stationary distribution of the population, that is, the average fraction of time the population spends in each of the *Z* + 1 states, is given by the eigenvector of the eigenvalue 1 of the transition matrix **M**^59^. Specially, the unitized eigenvector **Π** = [*π*_1_,⋅⋅⋅, *π*_*Z*+1_]^*T*^ is derived explicitly for *l* = 1, ⋅⋅⋅, *Z* + 1^60^:


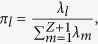


where *λ*_*l*_ = 1 if *l* = 1, and 
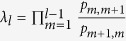
 otherwise.

In addition, another central quantity is the average cooperation level 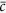, averaging over all possible states, weighted with the corresponding stationary distribution^61^,^62^. Accordingly, 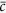 is computed as


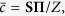


where **S** = [0,⋅⋅⋅, *Z*] is the vector of population states.

## Additional Information

**How to cite this article**: Chen, X. *et al.* Evolution of public cooperation in a monitored society with implicated punishment and within-group enforcement. *Sci. Rep.* **5**, 17050; doi: 10.1038/srep17050 (2015).

## Supplementary Material

Supplementary Information
