# Supplementary material for: Evolution of public cooperation in a monitored society with implicated punishment and within-group enforcement
Source: Sci Rep. 2015 Nov 24;5:17050. doi: 10.1038/srep17050 (PMC4657016; doi:10.1038/srep17050)
Supplement: Supplementary Information [file srep17050-s1.pdf]

Supplementary Information: Evolution of public cooperation in a monitored society with implicated punishment and within-group enforcement

Xiaojie Chen[1], Tatsuya Sasaki[2], Matjaž Perc[3,4,5]

1 School of Mathematical Sciences, University of Electronic Science and Technology of China, Chengdu 611731, China
2 Faculty of Mathematics, University of Vienna, 1090 Vienna, Austria
3 Faculty of Natual Sciences and Mathematics, University of Maribor, SI-2000 Maribor, Slovenia
4 Department of Physics, Faculty of Sciences, King Abdulaziz University, Jeddah, Saudi Arabia
5 Center for Applied Mathematics and Theoretical Physics, University of Maribor, SI-2000 Maribor, Slovenia

In the Supplementary Information, we first provide additional details regarding the stationary fraction of cooperators in infinite and finite populations (Fig. S1 and Fig. S2). Second, we explore the results regarding the stationary distribution and the average cooperation level for large peer punishment cost in finite populations (Fig. S3). Third, we investigate the effects of selection intensity on the stationary distribution and the average cooperation level in finite populations (Fig. S4). Fourth, we study the effects of mutation rates on the stationary distribution and the average cooperation level in finite populations (Fig. S5). Finally, we examine the effects of discounting factor of implicated punishment fine on the internal root of the gradient of selection both in infinite and finite populations (Fig. S6).

Stationary fraction of cooperators in infinite and finite populations for parameter variations

Figure S1 shows the stationary fraction of cooperators in infinite populations for different parameter combinations. We find that even if the implicated punishment fine and the within-group enforcement probability are increased greatly, there is still no interior equilibrium for small monitoring probability. But when the monitoring probability increases to a critical value, the interior equilibrium is then present. And the critical value

becomes smaller as the implicated punishment fine or the within-group enforcement probability increases. When the monitoring probability further increases, the value of the interior equilibrium decreases monotonically. However, when the parameter variations occur, there is no interior equilibrium for small implicated punishment fine, small within-group enforcement probability, or small group size. When these three parameters (d, q, and N) respectively reach the critical values, the interior equilibrium displays, and then its value decreases with increasing these parameters. Accordingly, the basin of attraction of the $x = 1$ steady state is enlarged. In addition, we see that with increasing the monitoring probability p, the implicated punishment fine d, the within-group enforcement probability q, or the group size N, the gradient of selection increases in the areas where $\dot{x} > 0$. But for a fixed value of p, d, q, or N, the gradient of selection can always reach the maximal values at an intermediate fraction of cooperators, which is smaller than $x = 1$.

Furthermore, we compute the interior root of $G(k)$ for finite populations in Fig. S2, which recovers to that for infinite populations in Fig. S1 when the population size $Z \to +\infty$. We find that the root's value for large Z is very close to that in infinite populations when the values of other parameter values are identical. Accordingly, the dependence of the root's value on the above four parameters (p, d, q, and N) is unchanged for finite populations. Altogether, increasing the monitoring probability, the implicated punishment fine, the within-group enforcement probability, or the group size can result in the emergence of the interior equilibrium of the system. And for large values of these four parameters, the basin of attraction of the full cooperation state can be greatly enlarged.

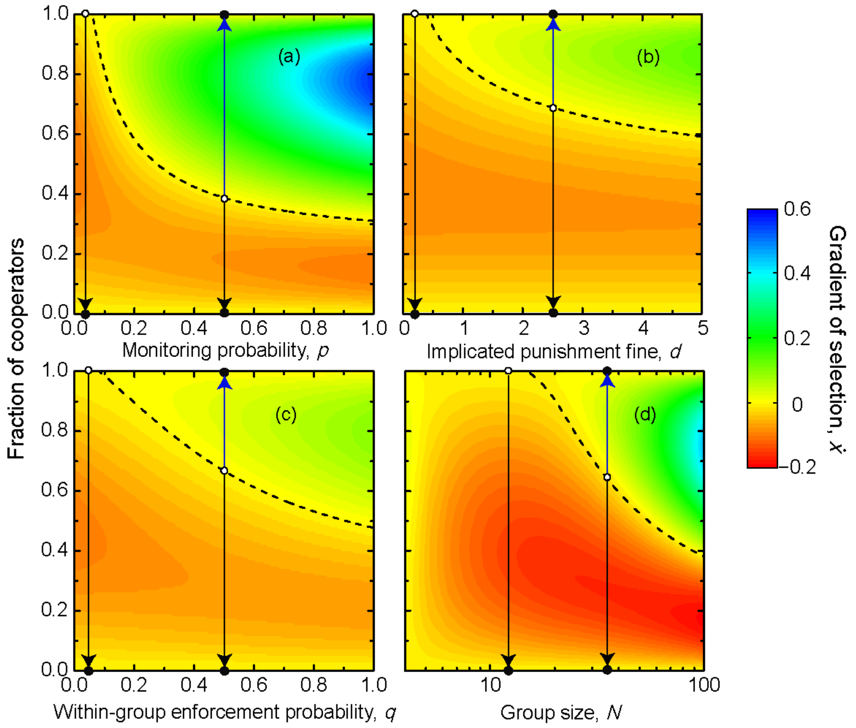

Figure S1. The stationary fraction of cooperators and the gradient of selection in infinite populations for parameter variations. The unstable internal equilibrium (if present) dividing the system into two basins of attraction is indicated by dash lines and also by open circles, and the cooperative basin of attraction is indicated by blue arrows. The stable boundary equilibrium is indicated by solid circles, while the unstable boundary equilibrium is indicated by open circles. The gradient of selection in the areas above the dash line is positive, while the gradient of selection in the areas below the dash line is negative. And the magnitude of the gradient of selection is shown using the red-green-blue scale indicated, and blue areas indicate parameters combination for which the fraction of cooperators increases faster than other areas. Parameters: $N = 5$, $r = 3$, $c = 1$, $d = 3.0$, $\alpha = 0.3$, $\beta = 1.0$, and $q = 0.9$ in (a); $N = 5$, $r = 3$, $c = 1$, $p = 0.5$, $\alpha = 0.3$, $\beta = 1.0$, and $q = 0.1$ in (b); $N = 5$, $r = 3$, $c = 1.0$, $p = 0.3$, $d = 1.0$, $\alpha = 0.3$, and $\beta = 1.0$ in (c); $r = 3$, $c = 1$, $p = 0.1$, $d = 1.0$, $\alpha = 0.3$, $\beta = 1.0$, and $q = 0.5$ in (d).

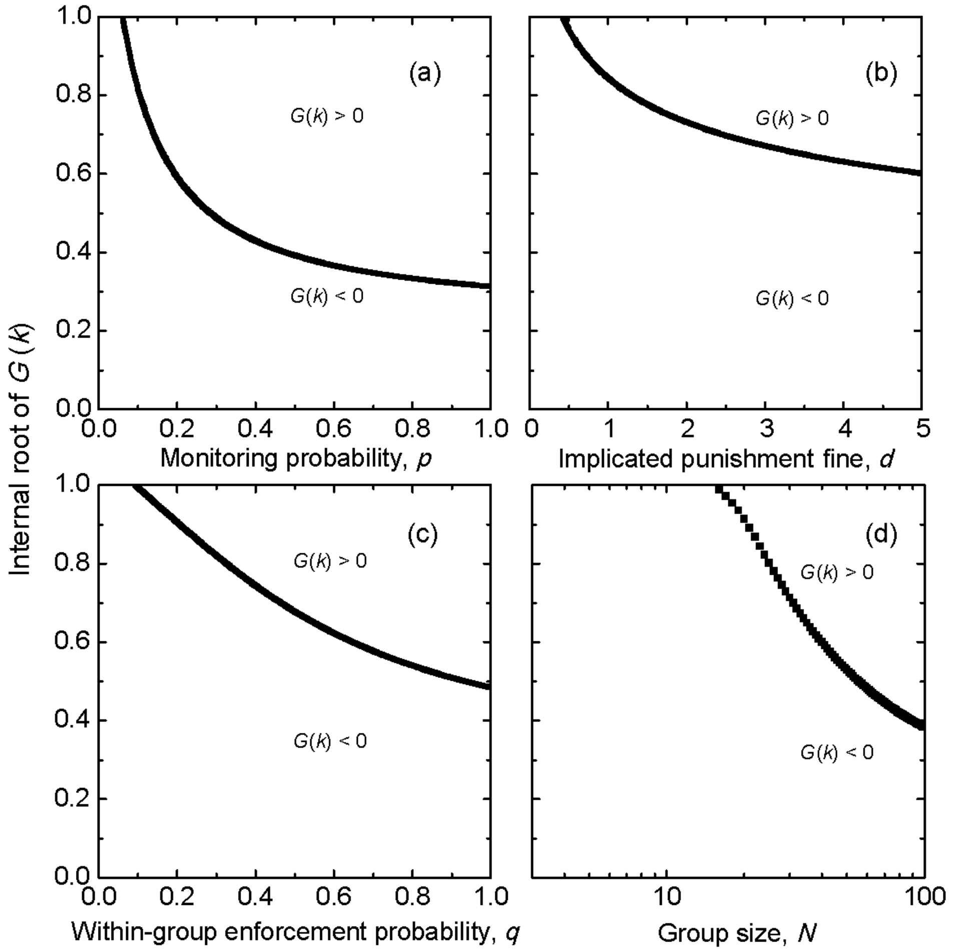

Figure S2. The internal roots of the gradient selection $G(k)$ in finite populations for parameter variations. The roots are normalized by the population size Z. Shown are the values of roots as a function of the monitoring probability p in (a), the implicated punishment fine d in (b), the within-group enforcement probability q in (c), and the group size N in (d), respectively. The values decrease with increasing these four parameters. Parameters: $Z = 200$, $N = 5$, $r = 3$, $c = 1$, $d = 3.0$, $\alpha = 0.3$, $\beta = 1.0$, and $q = 0.9$ in (a); $Z = 200$, $N = 5$, $r = 3$, $c = 1$, $p = 0.5$, $\alpha = 0.3$, $\beta = 1.0$, and $q = 0.1$ in (b); $Z = 200$, $N = 5$, $r = 3$, $c = 1$, $p = 0.3$, $d = 1.0$, $\alpha = 0.3$, and $\beta = 1.0$ in (c); $Z = 200$, $r = 3$, $c = 1$, $p = 0.1$, $d = 1.0$, $\alpha = 0.3$, $\beta = 1.0$, and $q = 0.5$ in (d).

Stationary distribution and average cooperation level for large peer punishment cost

Figure S3 shows the stationary distribution and the average cooperation level in the

presence of mutation $\mu = 0.01$ for large peer punishment cost. We still see that the population spends more time in states where cooperators prevail for larger monitoring probability, larger implicated punishment fine, or larger within-group enforcement probability. While the stationary distribution has a maximum at the full cooperation state for either a small group size or a large group size. We also observe that the average cooperation level increases with increasing the monitoring probability, the implicated punishment fine, or the within-group enforcement probability, even for a large peer punishment cost. When the monitoring probability, the implicated punishment fine, or the within-group enforcement probability is large, the cooperation level approaches one. But for a larger peer punishment cost, the critical values of these three parameters for a high cooperation level become larger. In addition, with increasing the group size the cooperation level first decreases to a small value which is close to zero, and it is kept there for an intermediate range of the group size. Then the cooperation level begins to increase when the group size exceeds the intermediate range, and finally reaches one at large group size.

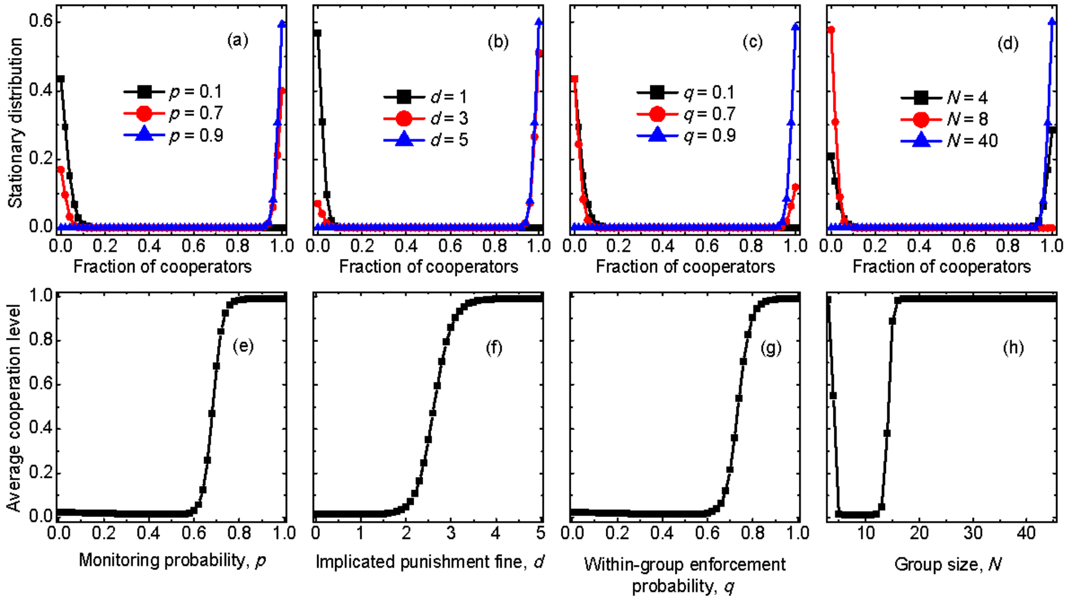

Figure S3. The stationary distribution and the average cooperation level for large peer punishment cost $\alpha = 0.5$. Top row depicts the stationary distribution in finite populations, and bottom row depicts the average cooperation level in the presence of mutation $\mu = 0.01$. Parameters: $Z = 50$, $N = 5$, $r = 3$, $c = 1$, $d = 1.0$, $\beta = 1.0$, and $q = 0.5$ in (a) and (e); $Z = 50$, $N = 5$, $r = 3$, $c = 1$, $p = 0.5$, $\beta = 1.0$, and $q = 0.5$ in (b) and (f); $Z = 50$, $N = 5$, $r = 3$, $c = 1$, $p = 0.5$, $d = 1.0$, and $\beta = 1.0$ in (c) and (g); $Z = 50$, $r = 3$, $c = 1$, $p = 0.5$, $d = 1.0$, $\beta = 1.0$, and $q = 0.5$ in (d)

and (h).

Effects of selection intensity on the stationary distribution and the average cooperation level

Figure S4 shows the stationary distribution and the average cooperation level for three different levels of selection intensity in the presence of $\mu = 0.01$. We see that for weak selection the stationary distribution has a maximum at the full defection state for small monitoring probability. But the system spends some time in either the full cooperator state or the coexistence state of cooperators and defectors. When the monitoring probability is large, the system spends most of the time in the full cooperation state for weak selection intensity, but it spends some time in the full defection state and the coexistence states of cooperators and defectors. While for moderate and large selection intensity, the system spends little time in the coexistence states, and it spends almost all the time in the states where defectors dominate for small monitoring probability. But when the monitoring probability becomes large, the system spends almost all the time in the states where cooperators prevail. In addition, these similar changes also occur when we investigate the influences of the implicated punishment fine, the within-group enforcement probability, and the group size on the stationary distribution for the three levels of selection intensity. Importantly, for these different levels of selection intensity, our main results are still valid: the system spends most of the time in states where cooperators prevail for a large monitoring probability, for large implicated punishment fine, for large within-group enforcement probability, or for either small or large group size.

In the bottom row, we plot the average cooperation level for the three levels of selection intensity at $\mu = 0.01$. We see that the average cooperation level increases with increasing the monitoring probability, the implicated punishment fine, or the within-group enforcement probability for different levels of selection intensity. And the cooperation level can reach the maximal value at an intermediate selection intensity for the intermediate range of the monitoring probability, the implicated punishment fine, or the within-group enforcement probability. This finding suggests that cooperation can be better promoted at moderate selection intensity. In addition, we see that with increasing the group size the cooperation level first decreases, then increases after reaching the minimal value for these three levels of selection intensity.

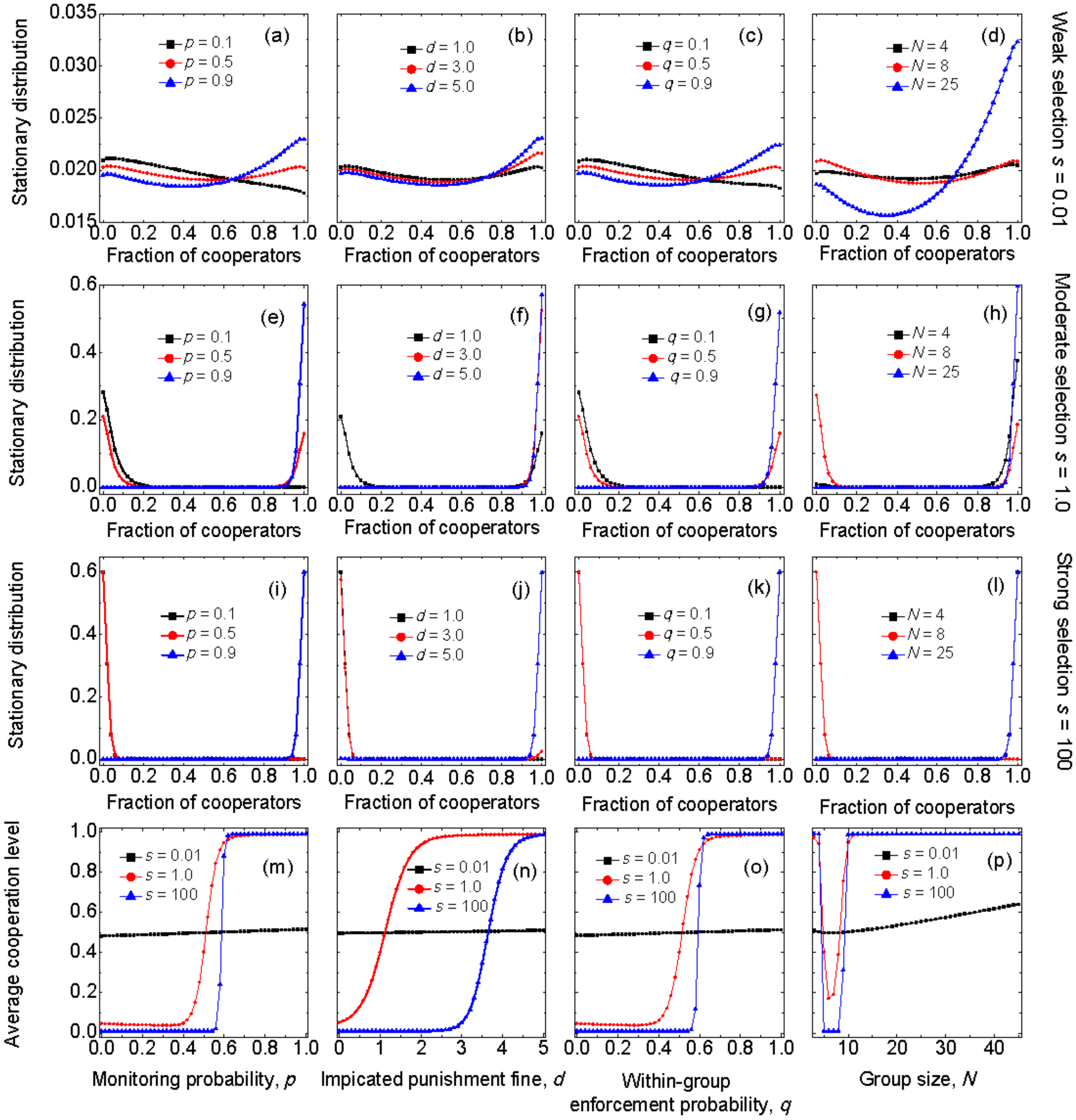

Figure S4. The stationary distribution and the average cooperation level for different levels of selection intensity. In the presence of $\mu = 0.01$, top row depicts the stationary distribution for weak selection intensity $s = 0.01$, second row depicts the stationary distribution of cooperation for moderate selection intensity $s = 1.0$, third row depicts the stationary distribution for strong selection intensity $s = 100$, and bottom row depicts the average cooperation level for these three levels of selection intensity. Parameters: $Z = 50$, $N = 5$, $r = 3$, $c = 1$, $d = 1.0$, $\alpha = 0.3$, $\beta = 1.0$, and $q = 0.5$ in (a, e, i and m); $Z = 50$, $N = 5$, $r = 3$, $c = 1$, $p = 0.5$, $\alpha = 0.3$, $\beta = 1.0$, and $q = 0.5$ in (b, f, j, and n); $Z = 50$, $N = 5$, $r = 3$, $c = 1.0$, $p = 0.5$, $d = 1.0$, $\alpha = 0.3$, and $\beta = 1.0$ in (c, g, k, and o); $Z = 50$, $r = 3$, $c = 1.0$, $p = 0.5$, $d = 1.0$, $\alpha = 0.3$, $\beta = 1.0$, and $q = 0.5$ in (d, h, l, and p).

Effects of mutation rates on the stationary distribution and the average cooperation level

Figure S5 shows the stationary distribution and the average cooperation level for different mutation rates. We see that for small mutation rate (top row), the system spends most of the time near the full cooperation state or the full defection state, leading to maxima of the stationary distribution at $Z = 0$ or $Z = k$. And for large monitoring probability, the stationary distribution will have a maxima at $Z = k$, which means that cooperation is greatly promoted accordingly. For large mutation rate (second row), we see that the stationary distribution has a maximum at a coexistence state which is close to the full defection state for small monitoring probability. While for intermediate monitoring probability, the stationary distribution keeps a maximum at the coexistence state, but also a second (smaller) maximum at another coexistence state which is close to the full cooperation state. For large monitoring probability, the stationary distribution has only a maximum at a coexistence state which is close to the full cooperation state. These results show that cooperation is promoted for large monitoring probability even for large mutation rate. When the mutation rate is further increased (third row), the stationary distribution only has a maximum centered around the intermediate values of the fraction of cooperators for each value of monitoring probability, and with increasing the monitoring probability the center moves towards the right, corresponding to a larger fraction of cooperators. Similarly, these changes about the stationary distribution occur when we investigate the effects of the implicated punishment fine, the within-group enforcement probability, and the group size for the three different mutation rates. But we find that our main results remain unchanged even at either a very small mutation rate or a very large mutation rate, that is, the system spends most of the time in a state where cooperators prevail for large monitoring probability, for large implicated punishment fine, for large within-group enforcement probability, or for either small or large group size.

In the bottom row, we show the average cooperation level for the three mutation rates. We see that the average cooperation level increases with increasing the monitoring probability, the implicated punishment fine, or the within-group enforcement probability. While for small mutation rate, it slowly increases with increasing the three parameters. In addition, the cooperation level is highest for small mutation rate when the monitoring probability, the implicated punishment fine, or the within-group enforcement probability is large. While the cooperation level is highest for very large mutation rate when the three parameters are small. We also see that with increasing the group size the cooperation level first decreases, then increases after reaching the minimal value for these three levels

of selection intensity.

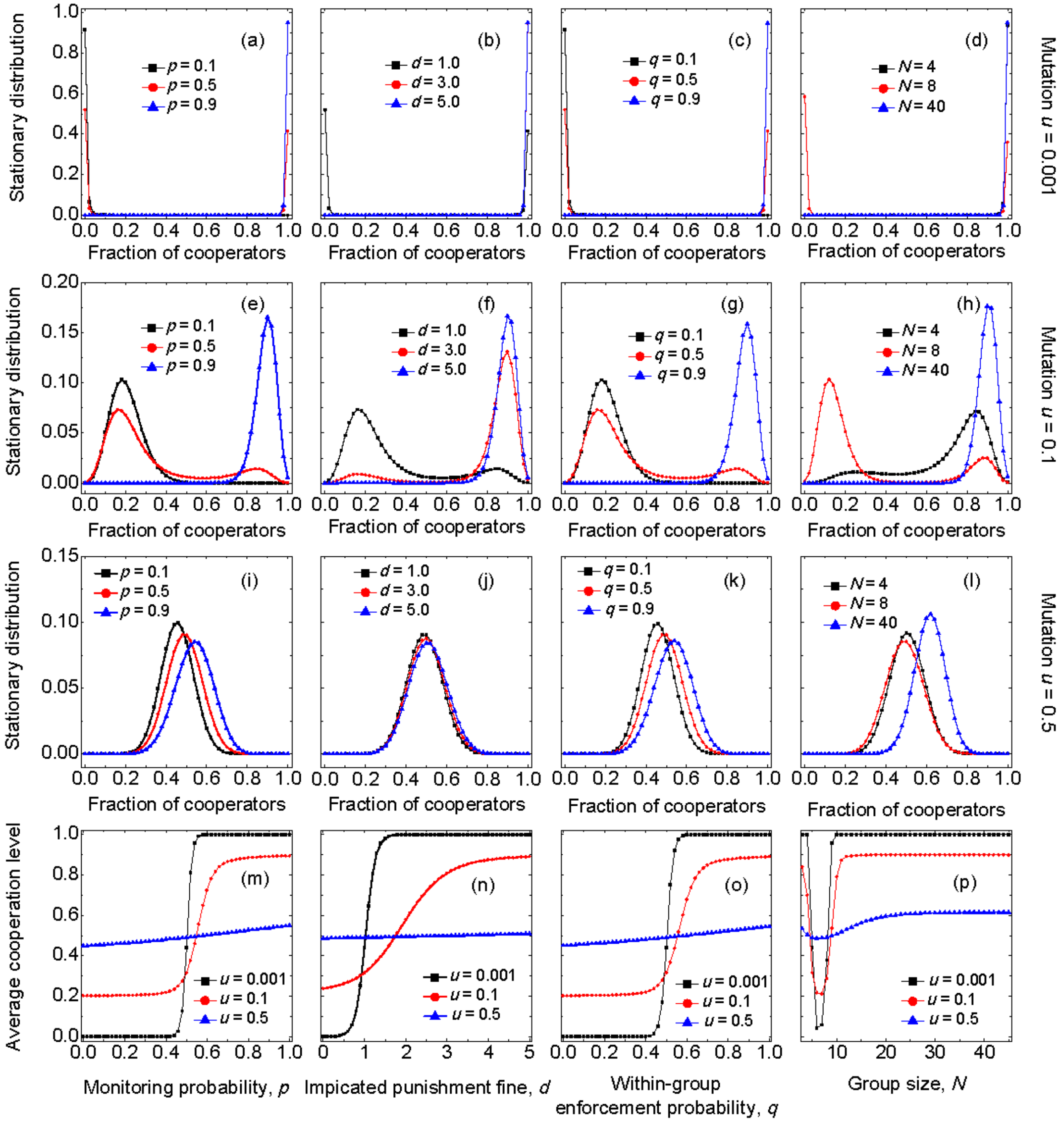

Figure S5. The stationary distribution and the average cooperation level for different mutation rates. For the intensity of selection $s = 2.0$, top row depicts the stationary distribution in finite populations for small mutation rate $\mu = 0.001$, second row depicts the stationary distribution of cooperation for large mutation rate $\mu = 0.1$, third row depicts the stationary distribution of cooperation for very large mutation rate $\mu = 0.5$, and bottom row depicts the average cooperation level for these three mutation rates. Parameters: $Z = 50$, $N = 5$, $r = 3$, $c = 1$, $d = 1.0$, $\alpha = 0.3$, $\beta = 1.0$, and $q = 0.5$ in (a, e, i and m); $Z = 50$, $N = 5$, $r = 3$, $c = 1$, $p = 0.5$, $\alpha = 0.3$, $\beta = 1.0$, and $q = 0.5$ in (b, f, j, and n); $Z = 50$, $N = 5$, $r = 3$, $c = 1.0$, $p = 0.5$, $d = 1.0$,

$\alpha = 0.3$, and $\beta = 1.0$ in (c, g, k, and o); $Z = 50$, $r = 3$, $c = 1.0$, $p = 0.5$, $d = 1.0$, $\alpha = 0.3$, $\beta = 1.0$, and $q = 0.5$ in (d, h, l, and p).

Effects of discounting factor of implicated punishment fine on the internal root of the gradient of selection

We consider a variant for the implementation of the implicated punishment, and assume that when a group of individuals are punished, each defector incurs a fine d, while each cooperator incurs a fine δd, where δ $(0 < \delta < 1)$ is a discounting factor of implicated punishment fine. Figure S6 shows the internal root of the gradient of selection as a function of the discounting factor δ in infinite and finite populations. We find that both in infinite and finite populations the internal root increases with increasing the discounting factor, which means that the basin of attraction of the full cooperation state becomes smaller and cooperators become disadvantageous with increasing the discounting factor.

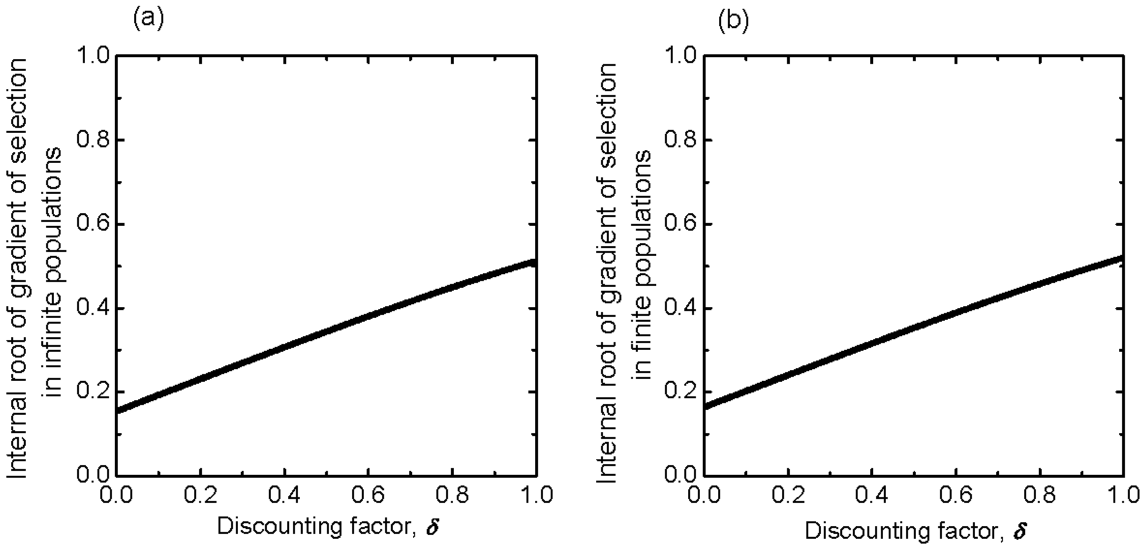

Figure S6. The internal root of the gradient selection in infinite (a) and finite (b) populations. In finite population the root is normalized by the population size Z. The root value increases with increasing the discounting factor δ. Parameters: $N = 5$, $r = 3$, $c = 1$, $d = 1.0$, $\alpha = 0.3$, $\beta = 1.0$, $p = 0.5$, and $q = 0.5$ in (a); $Z = 200$, $N = 5$, $r = 3$, $c = 1$, $d = 1.0$, $\alpha = 0.3$, $\beta = 1.0$, $p = 0.5$, and $q = 0.5$ in (b).
